# Community perspectives on mass malaria vaccine and drug administration in the Chittagong Hill Tracts, Bangladesh: a qualitative study

**DOI:** 10.1186/s12936-026-05999-6

**Published:** 2026-06-16

**Authors:** Md Fojle Rabby, Rupam Tripura, Ibrahim Khalil, Dewan Imtiaj Ahmed, Thomas J. Peto, Phaik Yeong Cheah, Md Amir Hossain, A K M Fazlur Rahman, Rasheda Samad, Rumana Rashid, Abdullah Abu Sayeed, Nicholas J. White, Nicholas P. J. Day, Arjen M. Dondorp, Lorenz von Seidlein, Bipin Adhikari, Md Abul Faiz

**Affiliations:** 1 Dev Care Foundation, Chittagong, Bangladesh; 2 Mahidol Oxford Tropical Medicine Research Unit (MORU), Faculty of Tropical Medicine, Mahidol University, Bangkok, Thailand; 3 Centre for Tropical Medicine and Global Health, Nuffield Department of Medicine, University of Oxford, Oxford, UK; 4 Centre for Injury Prevention and Research Bangladesh, Dhaka, Bangladesh

**Keywords:** Community perspectives, Mass vaccine, Mass drug administration, Malaria elimination, Qualitative research, Bangladesh

## Abstract

**Introduction:**

Malaria remains a major public health burden in Bangladesh, particularly in the Chittagong Hill Tracts (CHT), where indigenous and marginalized populations face persistent transmission risks. The Mass Vaccine and Drug Administration (MVDA) trial is currently being implemented to accelerate malaria elimination. MVDA is socially complex because it involves administering preventive interventions to largely asymptomatic populations in contexts where trust, prior experiences, and sociopolitical factors strongly shape acceptance. Little is known about community perspectives toward such interventions. This study explored local understandings of malaria, health-seeking behaviours, and perceptions of mass vaccine and drug administration in the CHT to inform community engagement and implementation of MVDA.

**Methods:**

A qualitative study collected data using focus group discussions (FGDs), in-depth interviews (IDIs), and key informant interviews (KIIs) in Lama and Alikadam sub-districts of Bandarban among community members with no prior exposure to formal research activities. Participants were purposively selected using a maximum variation sampling approach to capture diverse perspectives across socio-demographic and stakeholder groups. Participants included community members, village leaders, traditional healers, local pharmacists, village health workers, religious leaders, and local government representatives. A total of 105 participants from heterogeneous backgrounds participated in this study between September and October 2024. Data were transcribed, translated, and underwent primarily inductive thematic analysis led by a trained qualitative researcher, a Bengali non-native to the study site. Interpretation was conducted reflexively, considering the researcher’s positionality.

**Results:**

Malaria was recognized as a recurrent illness, particularly during the rainy season and among traditional farmers. While awareness of malaria symptoms and prevention had increased through non-governmental and government initiatives, health-seeking behaviour remained pluralistic. Communities relied on traditional healers and informal drug sellers before seeking formal care. Village health workers provided malaria diagnosis and treatment but faced challenges of inconsistent services (e.g., irregular supply of health materials). The recent COVID-19 experience seemed to shape vaccine perceptions, with mandates, and adverse event narratives contributing to mistrust. Knowledge of a malaria vaccine was limited, yet participants expressed conditional acceptance, indicating willingness to participate if interventions were perceived as safe, effective, and transparently communicated. MDAs were viewed more positively than mass vaccine administration(s) although adherence, particularly among children and adults was considered a challenge. Trust in institutions and historical political mistrust emerged as key determinants shaping community attitudes toward MVDA. These findings informed the design of community engagement strategies accompanying the MVDA intervention.

**Conclusions:**

The success of MVDA depends on achieving high population coverage, which is contingent on trust, legitimacy, and effective community engagement. In socially and politically complex settings such as the CHT, implementation must be grounded in context-specific strategies that are responsive to local social and cultural dynamics and informed by community perspectives.

**Supplementary Information:**

The online version contains supplementary material available at 10.1186/s12936-026-05999-6.

## Introduction

Bangladesh is one of the remaining malaria-endemic countries in South-East Asia, with an estimated 34% of its population at risk of infection [1]. Key determinants of malaria risk in Bangladesh include low socioeconomic status, age-related susceptibility, seasonal heterogeneity in transmission, inadequate knowledge of mosquito bite prevention, and proximity to mosquito breeding sites [2]. Malaria transmission persists in 13 of the country’s 64 districts, with the southeastern Chittagong Hill Tracts (CHT) bearing the highest burden, accounting for nearly 90% of malaria-related morbidity and mortality nationwide [3, 4]. Transmission in Bangladesh is largely seasonal, peaking during the rainy months from April to October [5]. *Plasmodium falciparum* is responsible for approximately 90% of infections [6].

Over the past two decades, Bangladesh has achieved substantial reductions in malaria incidence, with a 93% decline reported between 2008 and 2020. The national malaria elimination strategy has adopted a phased, district-wise approach based on transmission intensity but progress is constrained by challenges in prevention, timely diagnosis, and access to effective treatment [7–9]. The Bangladesh National Malaria Control Program (NMCP) has received six grants from the Global Fund to Fight AIDS, Tuberculosis and Malaria since 2006, implemented by the Ministry of Health and Family Welfare in collaboration with Bangladesh Rural Advancement Committee (BRAC). The program aimed to expand community-level access to diagnosis and artemisinin-based combination therapy (ACT) in hard-to-reach areas, distribute long-lasting insecticidal nets (LLINs) to achieve universal coverage in the three highest-burden CHT districts and strengthen epidemiological surveillance [10]. Malaria diagnosis and treatment are provided free of charge in Bangladesh, with community health workers delivering services at the local level [11]. Treatment-seeking behaviour among populations living in remote hill tracts continues to present a significant challenge echoing findings from previous studies in low resource settings [12–16].

Despite substantial reductions in malaria burden over the past decades, Bangladesh continues to face persistent malaria transmission in the CHT. Routine malaria control strategies—vector control, case management, and surveillance—have been effective nationally but appear insufficient to interrupt transmission, particularly among migrant and ethnic minority populations living in hard-to-reach areas. Aligning with the national malaria elimination target by 2030 [17], intensified strategies such as Mass Drug Administration (MDA) and Mass Vaccine Administration (MVA) are considered critical tools to accelerate malaria elimination by WHO [18–21]. Mass interventions using drug and/or vaccine administrations are often understood to provide population-level benefits while potentially raising concerns about individual autonomy [22]. At the same time, MDAs in low- to moderate-transmission settings are considered a promising strategy for malaria elimination [23]. Implementing these interventions (MDA and MVA) in remote and indigenous populations presents unique challenges and therefore requires intensive community engagement and trust-building to ensure participation among minority populations [24, 25]. It is critical to understand community members’ perspectives on malaria as a disease and on malaria interventions [13, 26]. Such baseline or formative community engagement also aligns with a people-centred approach to malaria elimination, including an equity-focused emphasis on reaching indigenous populations [27, 28].

A recent MDA trial in the Greater Mekong Subregion (GMS) has demonstrated the effectiveness of MDA in rapidly reducing transmission in a low transmission setting [19, 29]. However, the impact of MDA was time-limited as infections were re-imported into non-immune populations. MDA may accelerate malaria elimination when combined with other, more permanent interventions. The recent development of malaria vaccines, particularly R21/Matrix-M, represents an opportunity for malaria elimination. The integration of vaccination with MDA may provide a more sustained interruption of malaria transmission [30]. Mass Vaccine and Drug Administration (MVDA) is being evaluated in the Bandarban Hill District of Bangladesh.

The success of mass vaccine and antimalarial interventions depends on the population coverage, which is strongly influenced by social and cultural contexts, including community perspectives and perceptions of these interventions [24, 25, 31–35]. Coverage in mass interventions depends not only on biomedical efficacy but also on social legitimacy, trust, and perceived fairness, as conceptualized in frameworks of intervention acceptability [36]. In contested and marginalized settings, qualitative evidence is essential to understand how communities interpret, negotiate, and respond to MVDA, and to inform context-sensitive implementation strategies. The main objective of this study was to explore community perspectives on mass vaccine and drug administration for malaria to inform community engagement and the implementation of MVDA.

## Materials and methods

### Study design

**Fig. 1:**
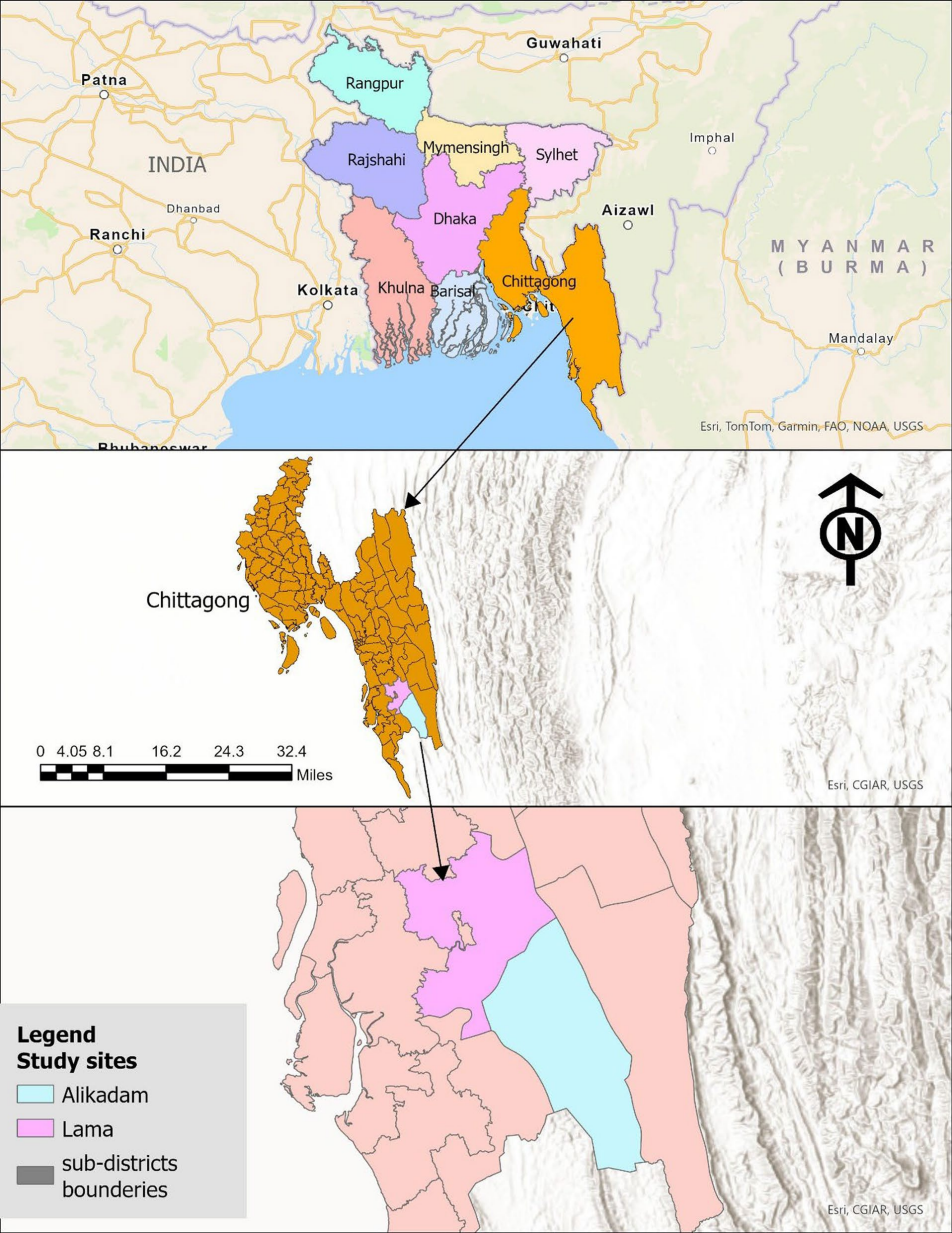
MVDA study sites are located in the Chittagong Hill Tracts within Lama and Alikadam sub-districts

This qualitative study employed a multi-method approach, including observations, focus group discussions, in-depth interviews, and key informant interviews, conducted across several phases of data collection and incorporating ethnographic elements through the interviewer’s repeated engagement and sustained presence at the study sites (Supplementary File 1: COREQ guideline). Data analysis and presentation were guided by a descriptive-interpretivist approach, underpinned by an interpretivist epistemology and a primarily inductive analytical orientation. The baseline study, conducted as a preparation for the MVDA trial, was carried out in villages within the Lama and Alikadam sub-districts of Bandarban, which serve as the MVDA field sites in Bangladesh (Fig. 1). In 2021–2022, the study areas in Bandarban had an Annual Parasite Incidence (API) ranging from 74 to 81 [7, 8]. While these are the highest transmission zones in Bangladesh, the API is much lower than in the World Health Organization (WHO) African Region with a recorded estimated API between 192 and 223 in 2024 [37]. The WHO recommends implementing MDA to reduce malaria transmission in settings with an API < 100 for *P. falciparum* and in all settings for *P. vivax* [37, 38].

### Mass vaccine and drug administration (MVDA) study

**Fig. 2:**
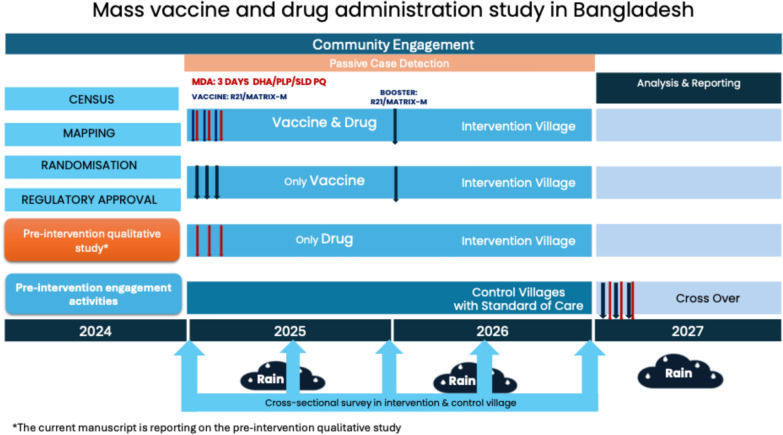
Community engagement and current study within the MVDA trial, Bangladesh

Mass Drug Administration (MDA) involves the provision of antimalarial drugs to entire populations regardless of infection status to reduce the parasite reservoir, while Mass Vaccine Administration (MVA) delivers vaccines at the population level to prevent infection. Mass Vaccine and Drug Administration (MVDA) combines these approaches to simultaneously reduce existing infections and prevent new ones. This is a cluster-randomized, open-label trial in 100 villages of the CHT, Bangladesh. This combined strategy is relatively novel, particularly in the Asian context, and aims to enhance and sustain malaria elimination efforts by integrating preventive and curative approaches. Based on our knowledge, this is the first study in Asia to evaluate the effectiveness of Mass Vaccine and Drug Administration (MVDA). The trial has a factorial design comparing four intervention arms: MVDA, Mass Vaccine Administration (MVA) alone, MDA alone, and the current standard of care (control), which includes the use of long-lasting insecticidal nets, early diagnosis, and treatment. Interventions consist of three rounds of vaccination and/or MDA (three doses), with a 12-month booster in the vaccine arm, while control villages will receive MVDA after 2 years (Fig. 2). The results of the trial will be reported elsewhere.

### Social and cultural context of study sites

The study villages are predominantly inhabited by ethnic minority communities, including the Mro, Tripura, Marma, and Tanchangya, alongside Bengali populations, with a recent increase in Bengali settlement. These communities are often mobile and reside in remote, hard-to-reach areas of the hill tracts. The CHT, comprising Rangamati, Khagrachari, and Bandarban districts, cover nearly 10% of Bangladesh’s land area and are characterized by significant ecological and cultural diversity, with around 12 indigenous groups collectively referred to as ‘Adivasi’ [39, 40]. Historically, local governance was organized through traditional Headman and ‘Karbari’ systems, which continue to play an important role in community decision-making.

The region has experienced longstanding socio-political tensions shaped by state-led settlement policies, displacement, militarization, and unresolved land rights issues, contributing to marginalization and mistrust among indigenous populations [41, 42]. Shifting (‘jhum’) cultivation, once central to indigenous subsistence, has been undermined by expanding commercial farming of crops such as tobacco [43]. These industrial pressures, coupled with unresolved land rights disputes, heavy military presence, and episodes of violence—including the abduction of ethnic minority populations, fuel long-standing grievances [44]. Indigenous organizations such as the Parbatya Chattagram Jana Samhati Samiti (PCJSS) aim to achieve regional autonomy, self-determination, and recognition of ethnic identity among indigenous peoples in CHT. These dynamics, along with geographic isolation and restricted access to certain areas, have influenced relationships with external actors, including government institutions and health services. Such historical and structural factors are important in understanding community perceptions of, and engagement with, externally introduced health interventions such as MVDA, where trust, legitimacy, and local acceptance are critical for participation.

### Participants

A diverse group of participants was recruited to explore the research questions in this study (Tables 1 and 2 and Supplementary File 2). Villages were purposively selected from the MVDA trial sites in Lama and Alikadam sub-districts to capture variation in geography, accessibility, and ethnic composition. First, local investigators and field research staff approached and invited potential respondents who could contribute to addressing the study question, based on their positions, roles, and the information they could provide, following the tenets of stakeholder analysis [45]. Second, from this initial list of stakeholders, a maximum variation sampling approach was employed to capture diverse perspectives across ages, genders, ethnic groups, villages, occupations, and hierarchical positions. This included participants from different ethnic groups, remote and relatively accessible villages, and individuals in both formal and informal community roles.

**Table 1:** Socio-demographics of the village leaders, religious leaders, schoolteachers and political figures (KIIs)

S.N	Age	Gender	Educational level	Occupation
1	50	M	Grade 0	Village leader and Farmer
2	55	M	Grade 5	Religious leader
3	30	M	Grade 8	Village leader and Farmer
4	35	M	Grade 12	School Teacher
5	70	M	Grade 0	Village leader and Farmer
6	50	M	Grade 0	Village leader and Farmer
7	33	M	Master level	School Teacher
8	65	M	Grade 0	Village leader and Farmer
9	50	M	Grade 0	Village leader and Farmer
10	40	M	Grade 0	Village leader and Farmer
11	50	M	Grade 10	Union council Chairman
12	60	M	Grade 5	Religious leader
13	38	M	Master level	School Teacher
14	50	M	Grade 0	Village leader and Farmer
15	45	M	Grade 0	Village leader and Farmer

**Table 2:** Socio-demographics of the village health workers, traditional healers and informal drug sellers, (IDIs)

S.N	Age	Gender	Educational level	Occupation
1	30	M	Bachelor level	Pharmacist
2	50	M	Grade 0	Traditional healer
3	35	M	Grade 10	Pharmacist
4	40	F	Grade 5	Village health worker
5	30	M	Grade 5	Village health worker
6	20	M	Grade 5	Village health worker
7	40	F	Grade 8	Village health worker
8	25	F	Grade 11	EPI worker
9	50	M	Grade 5	Pharmacist
10	30	M	Grade 5	Village health worker

The qualitative data collection methods included ten in-depth interviews with ten health providers, namely village health workers, traditional healers, local pharmacists, and staff from the Expanded Programme on Immunization (EPI). Fifteen key informant interviews were conducted with village leaders, religious leaders, schoolteachers, and union council chairmen. Eight focus group discussions (FGDs) were held with 80 residents from more than ten villages in Lama and Alikadam, stratified by gender, age, village and ethnicity (Supplementary File 3: Interview and FGD guide). Participants were eligible if they were adults residing in the study villages or stakeholders involved in local health and community leadership roles; no formal exclusion criteria were applied beyond the inability to provide informed consent. No financial or material incentives were provided for participation.

Recruitment ceased when thematic saturation was achieved across stakeholder categories, defined as the point at which no novel insights were recorded in further interviews with participants representing different socio-demographic groups (e.g., ethnicity, gender) and the study villages [46, 47]. Participants’ ages ranged from 18 to 72 years. The duration of interviews and discussions ranged from 45 to 60 min. None of the approached participants refused to participate in the interview, although a few rescheduled their appointments. While no participants formally refused participation, a few initially hesitated or rescheduled their appointments due to availability constraints.

### Positionality and reflexivity

MFR, a male Bengali social scientist with more than five years of field research experience, led the qualitative data collection for this study. MFR was involved in the MVDA project from its early stages, including initial stakeholder engagement at the national level. As a Bengali and Bangla-speaking researcher, MFR was linguistically aligned with the national context; however, he was perceived as an outsider within the ethnically diverse communities of the CHT.

Given the multilingual context of the study sites, local research assistants fluent in both Bangla and indigenous languages supported data collection by facilitating communication, translating interview guides, and assisting during interviews and discussions. While this enabled broader participation, the process of translation may have led to subtle losses of meaning or nuance in participants’ responses.

The socio-political context of the CHT, characterized by longstanding tensions and historical marginalization of ethnic minority groups, may have introduced power asymmetries between the researcher and participants. As a Bengali researcher, MFR was aware that his positionality could influence participants’ willingness to share openly, potentially contributing to social desirability bias or guarded responses.

To address these challenges, MFR adopted a reflexive and iterative approach throughout the research process. Drawing on his training in anthropology, he engaged in prolonged field presence, invested in building rapport and trust, and participated in everyday community activities, including sharing meals and spending extended time in village settings. These efforts aimed to reduce social distance and facilitate more open and contextually grounded discussions.

In addition, reflexivity was maintained during data analysis through regular discussions among the research team, allowing for critical examination of interpretations and minimizing individual bias in the analytical process. This process was supported by triangulation across data sources and peer debriefing within the research team. These positional dynamics were actively considered during analysis, particularly in interpreting expressions of trust, hesitancy, and participation, which may have been influenced by participants’ perceptions of the researcher and the broader research context.

### Data processing and analysis

Key informant interviews, in-depth interviews and focus group discussions were conducted within the study villages located in the Lama and Alikadam sub-districts of Bandarban between September and October 2024. All interviews were conducted in the respective local ethnic languages of the CHT by local bilingual staff (minority language and Bangla), audio recorded, transcribed verbatim in Bengali and subsequently translated into English for the purpose of thematic analysis. To ensure that meanings and interpretations were accurately retained, the lead interviewer, MFR, had cross-checked the transcripts against the audio files with the translator. MFR, a native Bengali speaker, served as the lead investigator and spent extended time in the field engaging with community members as part of the study team.

MFR as the lead interviewer, had extensive interactions with community members, although there was no prior relationship other than that established during the study. He also made observational notes during the field visits, which helped in understanding the social, cultural, and community dynamics and in contextualizing participants’ perspectives. A thematic synthesis was employed using Braun and Clarke’s (2006) six phases that included (1) familiarization, (2) coding, (3) theme generation, (4) theme review, (5) theme definition, and (6) reporting [48]. Although a hybrid coding approach was used, it was primarily inductive, allowing codes and themes to emerge from the data while being guided by the study objectives. Each transcript was examined line by line and coded in Taguette (Version 1.4.1–60-ga73a5e2) using a codebook developed for the initial interviews and subsequently adapted to reflect the emerging data and their patterns (MFR and BA). The codebook was further refined to address the specific research questions. For example, initial codes such as “fear of vaccine side effects,” “mistrust of government programmes,” and “previous COVID-19 experiences” were grouped into broader categories, which contributed to the development of higher-level themes such as vaccine hesitancy and conditional acceptance. Individual codes were consolidated to form overarching themes, each supported by illustrative quotations from the participants.

Following the completion of the coding, the landscape of the codes in terms of distribution, frequency, and relevance to the research question was discussed among the researchers (BA and MFR). Disagreements related to the codes and their interpretations were discussed with a third investigator (RT) and revised. Based on the discussion, major and minor themes were further refined and this informed the findings for this study (Supplementary File 4: Codebook). This iterative and collaborative process ensured that themes were both grounded in the data and analytically developed through team-based interpretation.

### Ethics approval

This study is part of the Mass Vaccine and Drug Administration (MVDA) study in Bangladesh and adheres to its established protocol. Ethical approval was obtained from the Bangladesh Medical Research Council (Reference: BMRC/NREC/2022–2025/268) and the Oxford Tropical Research Ethics Committee (OxTREC: 37-23) on 20 November 2023; Clinical Trial Registration Number: NCT06068530. Written informed consent was obtained from all respondents prior to their participation in the study. Participation was entirely voluntary, and it was clearly communicated that refusal to participate would not affect access to health services or involvement in the MVDA trial or related interventions. Given the context of prior vaccination mandates, particular care was taken to emphasize that participation in this qualitative study was independent of any government or trial-related requirements. To ensure confidentiality in small and close-knit village settings, identifying information was removed from transcripts, and participants were anonymized using non-identifiable descriptors in all reporting.

## Results

Respondents highlighted malaria’s impact alongside other seasonal illnesses related to poor sanitation and unsafe drinking water. Treatment-seeking behaviour was shaped by cost, distance, and a preference for traditional healers. Respondents shared mixed perceptions of the vaccine and malaria interventions, largely shaped by socio-political and historical mistrust. Respondents preferred communication and engagement in their languages through local leaders and youth group members. Illustrative quotes were provided where relevant (referenced by type of interview, stakeholder, gender, and village/’para’).

### Treatment seeking behaviour

Treatment-seeking behaviour reflected a pluralistic healthcare ecology, where individuals navigated between formal, informal, and traditional systems of care. Respondents recognized a wide range of conditions affecting their health including fever, typhoid, chronic cough, diabetes, and various skin conditions. Malaria was considered a recurrent and major health concern.

*“In our village, people get malaria and typhoid. During the hot season, they also suffer from headaches and fevers”*

*(IDI, male, Langi para)*

Some respondents shared their ailments such as back pain, knee pain and stomachache and headache. Most illnesses were perceived to be more prevalent during the rainy and winter months. Poor water supply and unsanitary conditions were thought to be the major cause of illness. Some respondents linked their ‘jhum’ cultivation practices to hazardous environmental conditions specifically exposure to mosquitoes.

*“Since we work in the hills, we often feel very hot and drink water, and that causes illness. Also, when we work in the jungle, we stay in unclean conditions and get bitten by mosquitoes, which leads to sickness.*

*(FGD, Male, Shilachondro para)*

The participants described treatment-seeking as a complex and dynamic process. Geographic remoteness emerged as a key challenge, with distance, cost, and poor road conditions acting as major barriers to accessing formal health services. These structural barriers not only limited access to biomedical care but also reinforced reliance on locally available and trusted alternatives. Living in remote areas as ethnic minorities further compounded these challenges, contributing to underlying political resentment.

Local traditional healers including indigenous practitioners known as *baidhya*, played a prominent role in community health practices, often being the point of first contact for care. The traditional and indigenous healers often treated illnesses with herbal medicine and spiritual interventions that also involved rituals and animal sacrifices. Their role extended beyond customary practice, reflecting established trust and cultural legitimacy within the community, particularly in contexts where formal health services were perceived as distant or unreliable. Trust in informal health services surpassed the trust in services provided by government institutions.

*“If we don’t get better, then we go to the traditional healer (baidhya), perform rituals, and make offerings of animals or birds. If that still doesn’t help, then we go to the hospital. Sometimes they survive, and sometimes they don’t”*

*(KII, male, Langring para)*

In addition, local medicine shops served as an alternative point of first contact for those seeking treatment. When a patient was severely ill and required hospitalization, Mro tribal beliefs forbade bringing the dead bodies back to the community.

Community residents had to cope with poor access to the urban areas where health centers were located, having to travel several hours on foot, and/or by boat during the rainy season.

*“Those from remote areas come on foot. Often, they come by boat. Sometimes, they walk halfway, then find a motorcycle and ride the rest of the way”*

*(IDI, male, Taw para)*

Treatment-seeking practices were shaped by an interplay of structural constraints, cultural norms, and trust, rather than solely by biomedical considerations, reflecting a context where access and legitimacy jointly influence healthcare decisions.

### Malaria knowledge and risk perception

Community understandings of malaria reflected a combination of biomedical knowledge, experiential familiarity, and normalized perceptions of risk shaped by repeated exposure. Malaria was perceived to be one of the most frequently experienced illnesses within their community, with the majority of individuals reporting that they had contracted the disease at least once in their lifetime. Knowledge of malaria was enhanced by recent efforts to provide information about malaria control interventions such as mosquito net distribution.

*“We came to know about malaria when other people started talking about it — like when government workers told us. Before, we hadn’t even heard the names of diseases like coronavirus or malaria. Now that government people are informing us, we’re learning about these illnesses. Also, organizations like BRAC give us mosquito nets, and from them, we learn a bit as well”*

*(KII, male, Langring para)*

This reflects the development of biomedical knowledge through external health interventions, which coexists with locally grounded understandings of illness. Malaria was perceived to persist as a recurrent health problem, particularly among those engaged in ‘jhum’ cultivation, whose prolonged exposure to mosquito-infested forest areas places them at heightened risk. Community participants knew that the higher burden of malaria in the past led to higher mortality. Respondents felt that health care had improved compared to the past, but they were worried about diseases and believed preventive measures were necessary.

*“Earlier, people were very scared that malaria could be fatal. But now they don’t believe that so much—they know there is treatment for malaria. If they get malaria, they will receive treatment. But we still warn them to properly hang mosquito nets. When they go to cut bamboo, we tell them to wear long-sleeved clothes”*

*(IDI, Female, Taw para)*

These accounts illustrate experiential knowledge, where repeated encounters with malaria shape perceptions of its severity and manageability. Over time, this has contributed to a normalization of malaria risk, where the disease is perceived as common and treatable rather than exceptional. Malaria was also perceived to peak during the rainy season, and in the hilly areas surrounding teak (‘Shegun’) plantations where mosquito density was considered to be higher. Poor sanitation, open defecation and water collection practices were perceived to be linked with mosquito prevalence.

While some community members were using mosquito nets regularly, some burned coils or semi-dried grass to generate smoke, and some believed smoking tobacco would repel mosquitoes. Some also believed that taking locally brewed rice wine would heat the blood and work as a natural repellent. These practices highlight the coexistence of biomedical prevention strategies with locally adapted and experiential practices, reflecting hybrid knowledge systems.

Community members attributed a unique cluster of signs and symptoms related to malaria that included weakness, chills, body pain, and dizziness. Among them, fever was most commonly reported as the defining characteristic of the disease.

*“When someone has malaria, they sometimes get headaches, body aches in the hands and legs, chills from time to time, vomiting or nausea. When these symptoms appear, we understand that it might be malaria”*

*(FGD, male and female, Shilachondro para)*

Symptom recognition was therefore largely grounded in experiential knowledge, enabling community-level identification of malaria in the absence of formal diagnosis. Despite improved access to treatment, some community members perceived malaria as potentially fatal, noting that delays of even a few days in treatment could result in death.

*“I wasn’t afraid at al [with long pause] …..If I died from malaria, … — I wasn’t scared. But without medicine, people can die from malaria”*

*(KII, male, Lulang para)*

This tension between normalization and perceived severity reflects a dual perception of malaria as both routine and potentially life-threatening. At the community level, diagnosis of malaria was often conducted by the village health workers, using Rapid Diagnostic Tests (RDTs) as a point-of-care diagnostic. However, there were instances where antimalarials were not available in the village due to stockouts, poor logistics, and inadequate coordination. These gaps in service delivery further shaped community reliance on experiential diagnosis and local practices, reinforcing the hybrid nature of malaria knowledge in this setting.

### Vaccine perceptions

Vaccine perceptions were shaped by a complex interplay of knowledge, prior experiences, and sociopolitical context, with COVID-19 serving as a key interpretive lens influencing attitudes toward MVDA. Community members had a wide range of perceptions about mass vaccine and drug administration related to their knowledge of these interventions and their potential impact. This knowledge influenced the likelihood of participating in the interventions.

Participants recalled the recent COVID-19 and measles vaccines, other routine childhood immunizations, vaccines for pregnant women, and rabies vaccines for dog bites. Knowledge of the malaria vaccine was limited, although they had heard about it in recent months.

*“I’ve heard the names of many vaccines, but I don’t know exactly what they’re for. Like the malaria vaccine, the coronavirus vaccine, and the vaccine for pregnant women”*

*(FGD, male and female, Krongoy para)*

This reflects limited biomedical understanding of vaccines, with awareness not necessarily translating into informed acceptance. Participants’ awareness of vaccines increased following the COVID-19 pandemic. Intensive vaccination campaigns and vaccine mandates also generated fear, skepticism, and misconceptions leading to widespread vaccine hesitancy. Some respondents perceived that vaccines were killing people.

*“It’s not that we’ve never heard about vaccines — we have. But we’ve also heard things like “people die after taking vaccines.” We’ve heard about vaccines, yes, but we haven’t seen them with our own eyes”*

*(KII, male, Langring para)*

Fears were shaped by multiple sources, including misinformation, circulating rumours, and lived or shared experiences, contributing to uncertainty and distrust. COVID-19 vaccine mandates were widespread as the state asked the residents to possess a vaccination card before being able to access public services such as schools, birth registration, public transport, markets, government services, and even residency. Some feared arrest for refusal, which obliged them to comply with the vaccine mandates. These experiences of coercion contributed to perceptions of limited autonomy, reinforcing distrust toward vaccination programmes.

Hesitancy and fear towards the COVID-19 vaccine were widespread among the community members, largely due to misinformation, fear of side effects, and a sense of coercion by the health system. Some respondents linked the vaccine to itching, burning sensations, scabies-like symptoms, which led to widespread concerns about vaccine safety.

*“After getting the vaccine, there was a burning sensation all over the body. After the vaccine was given, everyone experienced itching all over their bodies. The itching problem increased after the vaccination”*

*(FGD, female, Choto kolar jhiri)*

These concerns reflect fear grounded in adverse event narratives, which were often shared and amplified within the community, further shaping collective perceptions of vaccine risk. Despite government campaigns, including mandates, vaccination coverage remained poor, with many not taking the vaccine and the majority not completing the required doses.

*“No, many didn’t complete all the doses. Some were afraid, some didn’t come because they were busy, and others simply didn’t believe in the vaccine”*

*(KII, male, Kurukpata)*

In some study communities, mistrust of vaccines stemmed from a 2019 incident in which dogs reportedly died following a government rabies vaccination campaign. Moreover, community members expressed various fears regarding vaccination, including that vaccines cause death, shorten lifespan, or worsen pre-existing conditions such as hernia, and even population control.

*“The fear was that we might die after taking the vaccine. We once heard that there are too many people in Bangladesh, so the vaccine would be used to kill people and reduce the population. That’s why many didn’t take the vaccine. Also, we saw dogs being killed with vaccine, so we thought maybe the same would be done to people — like with the dogs. We heard that vaccines would be used to kill people to reduce the population. Honestly, I was very scared too. Sometimes people say strange things — I don’t really understand it myself.”*

*(KII, male, Upor panshi para)*

These narratives illustrate how historical events and rumours contributed to broader mistrust of external health interventions, extending beyond COVID-19 vaccination. Vaccine hesitancy was also prominent for Expanded Programme on Immunization (EPI) vaccines. Parents expressed concerns about vaccines that could kill their children. Community members also shared the discomfort associated with the vaccines, for instance, the sight of a syringe or needle and the fact that it triggers pain.

Some community members had heard about the mass malaria vaccination campaign through the MVDA team, but understanding was often incomplete. Many were unsure about who would receive the vaccine, and some questioned the need for vaccination when they were not ill.

*“We don’t know exactly who will take it, but we heard that whether someone has malaria or not, people will have to take this vaccine”*

*(FGD, male and female, Shilachondro para)*

This reflects a tension between preventive public health logic and local expectations of treatment, where interventions are typically sought only when symptomatic. Community members expressed reservations about accepting a vaccine only if it was demonstrably safe and effective. Some questioned the need for vaccination in the absence of illness, while others expressed outright refusal.

*“I personally won’t take the vaccine. If malaria is removed, surely some other disease will take its place, so let malaria stay in my village.”*

*(KII, male, Langring para)*

At the same time, a pattern of conditional acceptance emerged, where willingness to participate depended on perceived safety, effectiveness, and trust in those delivering the intervention. Many participants distinguished between the malaria and COVID-19 vaccines, expressing greater trust in a potential malaria vaccine, often linked to their lived experience with malaria. Participants emphasized the need for clear communication regarding the purpose and benefits of the vaccine. This suggests that experiential familiarity with malaria may enhance acceptance, provided that communication is transparent and trust is established.

### Attitudes toward mass drug administration

Attitudes toward MDA reflected a tension between preventive public health approaches (particularly as it involves taking antimalarials or vaccine when not ill) and locally grounded curative logics, shaping both understanding and participation. Linked to the routine care, most community members were aware of the antimalarials and recalled receiving medicines from BRAC and ‘Ekata’ health workers. Health workers often complained about non-compliance in taking antimalarials, particularly as patients did not adhere to the second or third dose when they already felt better.

*“I give it to them myself by going to their place, because they don’t want to take the medicine on their own. Some people even throw the medicine away and then say they’ve taken it. That’s why I make sure to give it myself. Many don’t want to take it because they say the medicine is as bitter as bitter gourd. And young children absolutely don’t want to take it — for them, we mix it in water and call it cold drinks to get them to take it.”*

*(IDI, male, Langring para)*

This highlights persistent compliance challenges, where treatment is often discontinued once symptoms subside, reflecting a symptom-driven approach to medication use. Alongside taking antimalarials when recommended by health workers, some community residents also applied traditional methods to recover from malaria. Home-brewed alcohol and turmeric powder were considered cures for malaria.

Community members reported hearing about the MDA intervention in their villages and received information primarily from the health workers. One of the prominent concerns was the rationale for taking antimalarials when they were not sick from malaria. Discussions with community residents enhanced their knowledge about malaria; however, understanding of the concept of ‘asymptomatic malaria’ and the rationale for MDA remained limited, with some clearly expressing reservations about participation.

*“That would be good. If there’s such medicine or vaccine that can prevent malaria, we’ll take it — but our consent must be taken first. “*

*(FGD, male and female, Shilachondro para)*

Community residents had mixed reactions to the concept of taking a combination of antimalarials and vaccine when they were not sick. This reflects confusion around asymptomatic treatment, where preventive interventions were difficult to reconcile with existing expectations of treatment being necessary only in the presence of illness. While some mixed the information they had received so far, some had a clear understanding of the interventions.

*“Yes, I’ve heard about it. I heard that it’s divided into four groups — one group will get only vaccines, another will get only medicine, another will get both vaccines and medicine, and another will receive mosquito nets. Then blood tests will be done to see which treatment works best — that’s what I heard.”*

*(KII, male, Nich panshi para)*

Some respondents expressed willingness to participate if the medicines or vaccines were proven effective. Others were hesitant, and some refused outright. Their main concerns were around the potential adverse effects and immediately linked them to their recent experience with the COVID-19 vaccine. Upon hearing such reactions, village leaders emphasized the need for clear and comprehensible messages on the concept of MVDA, and the need to build relationships and trust. These responses indicate a pattern of conditional acceptance, where willingness to participate depended on perceived effectiveness, safety, and trust in those delivering the intervention.

Some community members were willing to take antimalarials and the vaccine referring to their vulnerability to mosquito bites in their work at the forest and farms. They rationalized that taking these prophylactic interventions would prevent malaria, reflecting knowledge gained from state and NGO-led health education efforts. Attitudes toward MDA were shaped by the interplay between preventive methods and curative expectations, with participation influenced by understanding, trust, and prior experiences with health interventions.

### Community engagement preferences

Community engagement preferences reflected diverse, context-specific strategies shaped by local authority structures, communication practices, and evolving access to information.

### Role of traditional authority (*Karbari*)

Community members emphasized that village-level meetings were essential elements to reach everyone in the community, with the specific involvement of village leaders (*Karbari*), local representatives, health workers, and youths. One of the major suggestions was to tailor the messages in local languages to ensure that all community members understood the concept and rationale of the MVDA interventions. Trusted figures such as doctors, BRAC or ‘Ekata’ staff, and familiar community workers were deemed critical.

*Karbaris* were deemed to have a major influence in the community, as they had executive authority in maintaining governance, resolving community affairs, organizing meetings and coordinating activities such as ‘jhum’ cultivation, and managing emergencies. Community members primarily relied on the Karbari’s guidance on the majority of issues, including health-related campaigns, and the social affairs (e.g. social cohesion).

*“To be honest, brother, it's not possible to live in the village without the Karbari. The Karbari is absolutely essential; he is needed for everything. Without the Karbari, the village cannot function”*

*(FGD with youth, male, Dharmacharan para)*

This highlights the central role of traditional authority in legitimizing health interventions, where engagement through trusted local leadership is critical for community acceptance. Nonetheless, over the years, with the rise in education level, and the availability of the internet and technology, the influence of village leaders was perceived to be declining.

### Heterogeneity and tailored communication

Community residents also expressed heterogeneity in preferences for engagement activities, emphasizing the differences among villages and individuals within the community.

*“A meeting has to be held with the villagers. Not everyone in the village is the same — some believe, some don’t. Just like the five fingers of a hand aren’t the same, people aren’t either. If we hold a meeting using flip charts and posters, some will believe, and some still might not”*

*(KII, male, Upor panshi para)*

Other approaches recommended included mobile and interactive events using posters, flip charts, and videos. Community members emphasized the need to clearly explain how interventions would prevent malaria. These findings suggest that engagement strategies need to be flexible and tailored, recognizing variation in beliefs, understanding, and responsiveness within communities.

### Market spaces and tea stalls as communication hubs

Community members also recommended utilizing market areas for information dissemination, given that villagers did not have internet and thus often convened in the market to buy daily commodities. In addition, communicating with school children, teachers, army personnel, Union Parishad officials, clinic doctors, and pharmacists was deemed essential. Tea stalls were also identified as key venues for sharing information informally. These spaces function as informal communication hubs, enabling peer-to-peer information exchange and reinforcing community-level understanding of interventions.

### Youth and digital media

There was a growing recognition of increased use of social and digital media, particularly among younger populations.

*“We don’t have television, but everyone has mobile phones — in each household, there are two, three, even four phones”*

*(FGD, female, Choto kolar jhiri)*

This indicates emerging opportunities for digital engagement, particularly among younger community members, although access remains uneven across settings.

### Demand for adverse event monitoring

Even when participants expressed willingness to engage with interventions, there was a clear expectation for monitoring and managing adverse events. This reflects the importance of transparency and accountability in building trust, particularly in contexts where prior experiences have shaped concerns about intervention safety. Monitoring also requires a responsive approach that is anticipatory and adaptable to emerging issues and concerns, including rumours within the study sites.

### Shifting influence of authority

Nonetheless, with increasing education and access to technology, the influence of traditional leaders, particularly *Karbaris* was perceived to be gradually declining. This suggests a shifting landscape of authority, where traditional and modern forms of influence coexist and should be jointly considered in engagement strategies.

## Discussions

This qualitative study explored community perspectives on malaria, treatment-seeking behaviour, and attitudes towards MVDA in the CHT of Bangladesh—one of the least-studied populations and also the population with the highest malaria burden in Bangladesh. The population in the CHT is distinctive due to its socio-demographic characteristics, including historical and ongoing socio-political tensions with state institutions, which have also shaped responses to recent public health initiatives such as the COVID-19 vaccination campaign [41, 42].

Across findings, trust between population and the institution emerged as a central determinant shaping the acceptability and potential coverage of MVDA, particularly in a context marked by structural marginalization and historical mistrust of external interventions. These contextual challenges underscore the need for social science research as an integral component of community engagement to better understand local perspectives on malaria and proposed interventions. MVDA involves the administration of antimalarials and vaccines to individuals who are asymptomatic [26], with accompanying social, cultural, and biomedical complexities. Addressing these complexities requires sustained and embedded community engagement to build trust and relationships [24, 25, 27, 28].

Malaria was widely recognized as a recurrent illness, with common symptoms—including fever, chills, headache, and body pain—well known to community members [49]. Rather than simply reflecting biomedical awareness, community understandings of malaria were shaped by experiential knowledge and repeated exposure, where malaria was perceived as both routine and manageable.

Health-seeking behaviour reflected a pluralistic approach, where communities frequently consulted traditional healers and informal drug vendors prior to accessing formal health facilities [50]. This pluralistic healthcare ecology was driven not only by accessibility constraints but also by trust, where informal and traditional providers were often perceived as more reliable and culturally aligned than formal health systems. Access to formal healthcare was constrained by geographic isolation, high costs, and poor transportation infrastructure, underscoring the need to strengthen community-based services such as those provided by BRAC and Ekata health workers and ensure uninterrupted drug supply in remote areas [51]. Improved community-based health services have been established to enhance the diagnosis and management of malaria [29, 52, 53].

Perceptions of vaccination were strongly shaped by the recent COVID-19 campaign. COVID-19 functioned as a key interpretive lens through which malaria vaccination was understood, with recent experiences of mandates, misinformation, and adverse event narratives shaping current perceptions. Trust in vaccination was frequently undermined by fears of adverse effects, rumours, and experiences of coercion during the COVID-19 rollout, with some respondents associating vaccines with population control or external political agendas [31, 54]. These findings highlight how fear was not uniform but emerged through multiple pathways, including misinformation, perceived coercion, and shared narratives of adverse events. Cautious optimism emerged, with participants expressing willingness to accept a malaria vaccine if proven effective and supported by transparent, culturally sensitive communication strategies. This reflects a pattern of conditional acceptance, where participation depends on perceived safety, effectiveness, and trust in implementing actors.

Attitudes toward MDA revealed similar ambivalence. MDA highlighted a fundamental tension between preventive public health strategy and locally grounded curative expectations, where treatment is typically associated with symptomatic illness [55]. In addition, adherence seemed to be hindered by concerns about adverse effects, the unpleasant taste of tablets, and misconceptions about the need for completing treatment [24, 25, 34, 35].

### Implications for malaria elimination interventions, and programs

As countries embark on malaria elimination, remaining malaria hotspots are often concentrated in rural and remote regions where communities are geographically isolated, frequently inhabited by ethnic minorities, and unfamiliar with externally introduced interventions [56, 57]. Such settings are often distant from state institutions and systems of governance, and in the case of the CHT, communities are shaped by enduring historical and political grievances, including long-standing experiences of marginalization, displacement, and mistrust of government-led initiatives [42, 58]. These findings suggest that successful MVDA implementation requires community co-design of engagement strategies, ensuring that interventions are developed with, rather than for, local populations. Skepticism toward external programmes, including health initiatives, reflects broader concerns related to autonomy, cultural preservation, and justice [27, 59]. Transparent communication regarding adverse events and continuous monitoring systems are critical to addressing safety concerns and building trust. In these contexts, implementing previously successful interventions without a thorough understanding of local social and cultural dynamics can be counterproductive. Engagement strategies should therefore be culturally adapted, locally grounded, and sustained over time, rather than delivered as short-term campaign-based approaches. Early, formative research that foregrounds community perspectives on malaria, treatment-seeking behaviours, and interventions is therefore critical to informing politically and socially responsive, community-oriented engagement strategies [13, 26, 28, 57].

### Strengths and limitations

A strength of this study lies in its use of multiple qualitative methods—including FGDs, IDIs, KIIs, and observations (field notes)—across diverse participants (community members, village leaders, healers, health workers, political leaders and religious figures). This is the first study to explore the perspectives of CHT populations on malaria and MVDA interventions using a relatively large qualitative sample. This provides an in-depth understanding of community perspectives. The diversity of participants and the inclusion of multiple stakeholder groups enhance the theoretical transferability of the findings to similar malaria-endemic, politically complex, and hard-to-reach settings.

This study has limitations. The findings are not necessarily generalizable beyond the specific communities studied. Some views may have been influenced by social desirability and recall bias, particularly in group settings. Given that the study was conducted alongside the MVDA trial, there is a potential risk of social desirability bias, where participants may have provided responses aligned with perceived expectations of the research team. There is also a possibility of therapeutic misconception, where participants may have conflated research activities with direct health benefits, particularly in the context of ongoing intervention trials. Although efforts were made to ensure accurate translation, subtle nuances in meaning may have been lost during transcription and translation from local languages to Bengali and English. In addition, key informant interviews with leadership figures were predominantly conducted with male participants, which may have limited the representation of gendered perspectives in leadership roles. Collating perspectives from a broad group of respondents, together with field notes based on observation and extended time spent with community members, may have mitigated potential biases.

## Conclusions

Malaria remains a major health concern in the CHT of Bangladesh, shaped by environmental exposure as well as longstanding social, cultural, and structural factors. While communities recognize the burden of malaria and the need for effective control, perceptions of MVDA were heterogeneous and marked by cautious receptiveness. Concerns related to past mistrust, fear of side effects, and logistical barriers continue to influence acceptability and participation. For malaria elimination programmes, these findings underscore that in politically sensitive and ethnically diverse settings such as the CHT, biomedical tools alone are insufficient; legitimacy, transparency, and trust-building are central determinants of population-level impact. If proven to be safe and effective, future programmatic rollout of MVDA will require context-specific, community-centered, and sustained engagement strategies, including strengthening village health workers, enhancing participatory health education, ensuring transparent communication, promoting adherence to drug regimens, and addressing structural barriers such as transportation and access to care. MVDA strategies in the CHT must therefore integrate biomedical interventions with long-term, trust-building community engagement to ensure equitable and effective malaria elimination.

## Supplementary Information

Supplementary material 1. COREQ Guideline.

Supplementary material 2. Table showing socio-demographics of FGD participants.

Supplementary material 3. Interview guide for IDIs, FGDs and KIIs.

Supplementary material 4. Interview guide for IDIs, FGDs and KIIs.

## Data Availability

Due to concerns regarding participant confidentiality and the potential risk of identification, the data underlying this study cannot be made publicly available. However, data may be accessed upon reasonable request to the Mahidol Oxford Tropical Medicine Research Unit (MORU) Data Access Committee, in accordance with the Unit’s data access policy (http://www.tropmedres.ac/data-sharing).

## Abbreviations

CHT: Chittagong Hill Tracts
EPI: Expanded Programme on Immunization
FGD: Focus group discussion
IDI: In-depth interview
KII: Key informant interview
MDA: Mass Drug Administration
MVA: Mass Vaccine Administration
MVDA: Mass Vaccine and Drug Administration
RDT: Rapid Diagnostic Test
