# Supplementary material for: Community perspectives on mass malaria vaccine and drug administration in the Chittagong Hill Tracts, Bangladesh: a qualitative study
Source: Malar J. 2026 Jun 16;25:244. doi: 10.1186/s12936-026-05999-6 (PMC13312631; doi:10.1186/s12936-026-05999-6)
Supplement: Supplementary file 2 — Supplementary material 2. Table showing socio-demographics of FGD participants. [file 12936_2026_5999_MOESM2_ESM.docx]

# Community perspectives on mass malaria vaccine and drug administration in the Chittagong Hill Tracts, Bangladesh: a qualitative study

Md Fojle Rabby¹, Rupam Tripura²^,3^, Ibrahim Khalil¹, Dewan Imtiaj Ahmed¹, Thomas J Peto²^,3^, Phaik Yeong Cheah²^,3^, Md Amir Hossain¹, A. K. M. Fazlur Rahman³, Rasheda Samad¹, Rumana Rashid¹, Abdullah Abu Sayeed¹, Nicholas J White²^,3^, Nicholas PJ Day²^,3^, Arjen M Dondorp²^,3^, Lorenz von Seidlein²^,3^, Bipin Adhikari²^,3*^, Md Abul Faiz^1, 2^

¹Dev Care Foundation, Chittagong, Bangladesh

²Mahidol Oxford Tropical Medicine Research Unit (MORU), Faculty of Tropical Medicine, Mahidol University, Bangkok, Thailand

^3^Centre for Tropical Medicine and Global Health, Nuffield Department of Medicine, University of Oxford, Oxford, UK

*Bipin@tropmedres.ac

**Supplementary File 2**

**Table 1**: Socio-demographics of the community people including Male, Female and youth, (FGDs)

| S.N. | Age | Gender | Educational level | Occupation |
| --- | --- | --- | --- | --- |
| 1 | 27 | F | Grade 0 | Housewife |
| 2 | 60 | F | Grade 0 | Housewife |
| 3 | 40 | F | Grade 0 | Housewife |
| 4 | 35 | F | Grade 0 | Housewife |
| 5 | 40 | F | Grade 0 | Housewife |
| 6 | 25 | F | Grade 0 | Housewife |
| 7 | 55 | F | Grade 0 | Housewife |
| 8 | 50 | F | Grade 0 | Housewife |
| 9 | 38 | F | Grade 0 | Housewife |
| 10 | 60 | M | Grade 0 | Farmer |
| 11 | 45 | M | Grade 0 | Farmer |
| 12 | 30 | M | Grade 0 | Farmer |
| 13 | 40 | M | Grade 0 | Farmer |
| 14 | 60 | M | Grade 0 | Farmer |
| 15 | 50 | F | Grade 0 | Housewife |
| 16 | 35 | F | Grade 0 | Housewife |
| 17 | 65 | M | Grade 0 | Farmer |
| 18 | 50 | M | Grade 0 | Farmer |
| 19 | 25 | M | Grade 1 | Farmer |
| 20 | 40 | M | Grade 0 | Farmer |
| 21 | 30 | M | Grade 5 | Farmer |
| 22 | 35 | M | Grade 0 | Farmer |
| 23 | 40 | F | Grade 0 | Housewife |
| 24 | 35 | F | Grade 0 | Housewife |
| 25 | 30 | F | Grade 0 | Housewife |
| 26 | 40 | F | Grade 0 | Housewife |
| 27 | 50 | F | Grade 0 | Housewife |
| 28 | 30 | F | Grade 0 | Housewife |
| 29 | 26 | F | Grade 0 | Housewife |
| 30 | 45 | F | Grade 0 | Housewife |
| 31 | 50 | F | Grade 0 | Housewife |
| 32 | 60 | F | Grade 0 | Housewife |
| 33 | 42 | F | Grade 0 | Housewife |
| 34 | 45 | F | Grade 0 | Housewife |
| 35 | 25 | F | Grade 0 | Housewife |
| 36 | 70 | M | Grade 0 | Farmer |
| 37 | 18 | M | Grade 5 | Farmer |
| 38 | 60 | F | Grade 0 | Housewife |
| 39 | 30 | M | Grade 5 | Farmer |
| 40 | 40 | F | Grade 0 | Housewife |
| 41 | 50 | F | Grade 0 | Housewife |
| 42 | 28 | F | Grade 0 | Housewife |
| 43 | 40 | F | Grade 0 | Housewife |
| 44 | 60 | F | Grade 0 | Housewife |
| 45 | 32 | F | Grade 0 | Housewife |
| 46 | 50 | F | Grade 0 | Housewife |
| 47 | 50 | F | Grade 0 | Housewife |
| 48 | 55 | F | Grade 0 | Housewife |
| 49 | 25 | M | Grade 8 | Farmer |
| 50 | 20 | M | Grade 5 | Farmer |
| 51 | 25 | M | Grade 10 | Farmer |
| 52 | 26 | M | Grade 5 | Farmer |
| 53 | 20 | M | Grade 5 | Farmer |
| 54 | 30 | M | Grade 5 | Farmer |
| 55 | 65 | M | Grade 0 | Farmer |
| 56 | 50 | M | Grade 0 | Farmer |
| 57 | 30 | M | Grade 5 | Farmer |
| 58 | 45 | M | Grade 0 | Farmer |
| 59 | 50 | F | Grade 0 | Housewife |
| 60 | 45 | F | Grade 0 | Housewife |
| 61 | 20 | M | Grade 0 | Farmer |
| 62 | 20 | M | Grade 0 | Farmer |
| 63 | 18 | M | Grade 0 | Farmer |
| 64 | 18 | M | Grade 5 | Farmer |
| 65 | 20 | M | Grade 0 | Farmer |
| 66 | 25 | M | Grade 0 | Farmer |
| 67 | 20 | M | Grade 0 | Farmer |
| 68 | 23 | M | Grade 0 | Farmer |
| 69 | 20 | M | Grade 0 | Farmer |
| 70 | 22 | M | Grade 0 | Farmer |
| 71 | 30 | F | Grade 5 | Housewife |
| 72 | 40 | F | Grade 0 | Housewife |
| 73 | 20 | F | Grade 5 | Housewife |
| 74 | 30 | M | Grade 0 | Farmer |
| 75 | 25 | M | Grade 0 | Farmer |
| 76 | 40 | M | Grade 0 | Farmer |
| 77 | 50 | F | Grade 0 | Housewife |
| 78 | 60 | F | Grade 0 | Housewife |
| 79 | 65 | F | Grade 0 | Housewife |
| 80 | 30 | M | Grade 0 | Farmer |
