# Supplementary material for: Community perspectives on mass malaria vaccine and drug administration in the Chittagong Hill Tracts, Bangladesh: a qualitative study
Source: Malar J. 2026 Jun 16;25:244. doi: 10.1186/s12936-026-05999-6 (PMC13312631; doi:10.1186/s12936-026-05999-6)
Supplement: Supplementary file 3 — Supplementary material 3. Interview guide for IDIs, FGDs and KIIs. [file 12936_2026_5999_MOESM3_ESM.docx]

# Community perspectives on mass malaria vaccine and drug administrations in the Chittagong Hill Tracts region, Bangladesh

Md. Fojle Rabby¹, Rupam Tripura²^,3^, Ibrahim Khalil¹, Dewan Imtiaj Ahmed¹, Thomas J Peto²^,3^, Phaik Yeong Cheah²^,3^, Md Amir Hossain¹, A. K. M. Fazlur Rahman³, Rasheda Samad¹, Rumana Rashid¹, Abdullah Abu Sayeed¹, Nicholas J White²^,3^, Nicholas PJ Day²^,3^, Arjen M Dondorp²^,3^, Lorenz von Seidlein²^,3^, Bipin Adhikari²^,3*^, Md. Abul Faiz^2,4^

¹Dev Care Foundation, Chittagong, Bangladesh

²Mahidol Oxford Tropical Medicine Research Unit (MORU), Faculty of Tropical Medicine, Mahidol University, Bangkok, Thailand

^3^Centre for Tropical Medicine and Global Health, Nuffield Department of Medicine, University of Oxford, Oxford, UK

^4^Centre for Injury Prevention and Research (CIPRB), Dhaka, Bangladesh

[*Bipin@tropmedres.ac](mailto:*Bipin@tropmedres.ac)

**Supplementary File 3**: MVDA baseline Guidelines for In-Depth Interviews (IDI), Focus Group Discussions (FGD), and Key Informant Interviews (KII)

| **Section- A: Socio Demographic Information** | | | | |
| --- | --- | --- | --- | --- |
|  | | | | Respondent ID:  Village code:  Sex:  Ethnicity:  Age:  Education:  Occupation: |
| **Section-B: Health problems and health care seeking behavior in general** | | | | |
|  | | | | How are you all doing these days? Any of you or your families are not well these days? (If none of them say no one is sick, then ask how about in the past, for example, one month ago etc.)  In your opinion, what are the most common health problems in your village? Why?  Do people in this village tend to suffer from this illness at particular times of the year? If so, when?  If somebody feels sick in your village, where do they normally go for treatment?  Why do people tend to go to XXX and YYY for their treatment?  Do people go to different place to seek treatment during different times of the year? If not, why not?  Are there any reasons why someone in this community would not seek treatment? (e.g. When is it too expensive? how far is too far? the severity of illness they consider to seek treatment, are there concerns around the quality of health center?) |
| **Section -C: Knowledge, Attitude and Practices towards Malaria** | | | | |
|  | | | Do you know or have you ever heard about malaria? Are there any other terms for malaria which people in this village call it?  What are the symptoms of malaria?  How do you know if somebody has malaria?  How big is a malaria problem in your village? Why?  What are the initiatives that people take to prevent malaria? (probe: Mosquito net , Burning the wood, By creating smoke, wear sleeved cloths and ETC)  Do you think that a healthy person (who is walking around and working normally) can have malaria parasite in his/her body? Why (for both yes and no answers)?  If yes, do you think are these people dangerous? Why (for both yes and no answers)?  What should we do with these people then? Why?  Do people in this village tend to suffer from malaria at particular times of the year? If so, when?  If someone in your village thinks he/she has malaria, where does he/she normally go for treatment?  Why do people tend to go to XXX and XXX for their treatment? (probe: around financial, traditional beliefs, proximity to home, trust in service etc.)  Do people go to the same place to seek treatment for malaria no matter what time of the year it is? If not, why not.  Are there reasons why someone in this village would not seek treatment? (e.g. at what point do most people find cost prohibitive, how far is too far, how sick do people consider they need to be to seek treatment)  Do people also visit more than one place if they have malaria? Can you explain where and who decides? (probe for household decision making dynamics) | |
| **Section- D: Knowledge, attitude and practices towards Mass Vaccine Administration** | | | | |
|  | | Do you know or have you ever heard about vaccine?  If yes, from whom and how did you hear about it (can you tell me few vaccines you know?)  How did you know about the vaccines? (where did you hear about it?)  What method do you prefer to get the information? (probe: Flipchart, Poster, leaflet, video and meeting)  Have you or your family members/village people taken any vaccine such as childhood routine vaccines or more recently COVID vaccine?  Did you feel any hesitation to take the vaccines? (for you and for your child or any other family members, if so, why?)  If you took the vaccine, what motivated you? (For childhood vaccines and for yourself, e.g. during the pandemic?)  If you or your family member did not take the vaccine, could you share what was the major concern for you or your family members?  Do you know or have you heard about mass vaccination administration project in your village?  Can you tell us what this project is about? Why is this project here?  Do you think malaria can be eliminated from the village (using the vaccine for malaria)? Why and how?  One way to eliminate malaria is to take 1.) Mass vaccine and Drug, 2.) Only Vaccine, 3.) Only drug and 4.) Standard of care by all residents in the village regardless of whether they are sick or not, do you think people will take medicine even when they are not sick? And how about yourselves?  To get rid of malaria from your village, we need to check blood including taking of vaccines and drugs by all community members. Do you think people will agree to these procedures? Why? | | |
| **Section- E: Knowledge, Attitude and practices towards Mass Drug Administration** | | | | |
|  | Have you heard about antimalarial medicine?  If yes, how did you hear about it?  Have you or your family members taken anti-malarias in the past? What did you (or your family members) think of the antimalarial? (probe: are they tolerable, safe, easy to take?)  If yes, what was the motivation to take the antimalarial drug?  If no, what was the reason for not taking the antimalarial?  One way to eliminate malaria from your community is to provide antimalarial to all the community members regardless of whether they have malaria or not. Do you agree with this idea?  Would you take antimalarial even if you are not sick? If yes and no, why?  Have you heard of mass anti-malarial administration in your community? (if so, can you give examples where and how you heard about it?)  Can you tell me how would you like to know the information about the mass antimalarial administration or medicine for malaria? (probe: flipchart, poster, leaflet, video, meeting, health center, health staff) | | | |
| **Section- F: Information disseminating** | | | | |
|  | Do you think all villagers in your village know or have heard about the mass vaccine and drug administration project? Why?  Someone might have not have known or heard about this project, what can we do to inform them about our research project?  How can we make sure that all people can get the information in the village? | | | |
