# Supplementary material for: Community perspectives on mass malaria vaccine and drug administration in the Chittagong Hill Tracts, Bangladesh: a qualitative study
Source: Malar J. 2026 Jun 16;25:244. doi: 10.1186/s12936-026-05999-6 (PMC13312631; doi:10.1186/s12936-026-05999-6)
Supplement: Supplementary file 4 — Supplementary material 4. Interview guide for IDIs, FGDs and KIIs. [file 12936_2026_5999_MOESM4_ESM.docx]

# Community perspectives on mass malaria vaccine and drug administrations in the Chittagong Hill Tracts region, Bangladesh

Md. Fojle Rabby¹, Rupam Tripura²^,3^, Ibrahim Khalil¹, Dewan Imtiaj Ahmed¹, Thomas J Peto²^,3^, Phaik Yeong Cheah²^,3^, Md Amir Hossain¹, A. K. M. Fazlur Rahman³, Rasheda Samad¹, Rumana Rashid¹, Abdullah Abu Sayeed¹, Nicholas J White²^,3^, Nicholas PJ Day²^,3^, Arjen M Dondorp²^,3^, Lorenz von Seidlein²^,3^, Bipin Adhikari²^,3*^, Md. Abul Faiz^2,4^

¹Dev Care Foundation, Chittagong, Bangladesh

²Mahidol Oxford Tropical Medicine Research Unit (MORU), Faculty of Tropical Medicine, Mahidol University, Bangkok, Thailand

^3^Centre for Tropical Medicine and Global Health, Nuffield Department of Medicine, University of Oxford, Oxford, UK

^4^Centre for Injury Prevention and Research (CIPRB), Dhaka, Bangladesh

[*Bipin@tropmedres.ac](mailto:*Bipin@tropmedres.ac)

**Supplementary File 4**: codebook generated from Taguette

| Name of the code | Definition | Highlights (frequency) |
| --- | --- | --- |
| Treatment seeking | The actions people take when they face any illness, including how they recognize symptoms, the decisions they make about where to seek care, the use of home remedies or traditional healers, visiting clinics or hospitals, and their overall response health seeking behavior | 135 |
| Vaccine | People' existing knowledge about vaccine | 105 |
| Covid-19 | People's attitudes and perceptions regarding COVID-19 and the COVID-19 vaccine reflect various levels of awareness, trust, and acceptance. | 73 |
| MVDA | People's KAP Mass vaccine and Drug administration study in Bangladesh | 67 |
| Engagement | Strategies for effectively engaging and involving community members in the MVDA study based on their opinion | 60 |
| Fear of vaccine | People's fear regarding vaccination | 58 |
| Antimalarial | Medications used for the treatment of malaria | 58 |
| Common diseases | The most common disease currently affecting the population. | 55 |
| Traditional healers | Person who uses traditional knowledge, medicinal plants, and cultural practices to treat illnesses within the community. | 52 |
| Information and communication | The methods by which people share and exchange knowledge or messages, as well as how they receive information from outside the village. | 45 |
| Precautions | The measures people are taking to protect themselves from malaria. | 41 |
| Village leader | The village head is the community leader responsible for disseminating information among villagers and serving as an influential figure within the community. | 39 |
| Health workers | The village health worker is responsible for providing malaria treatment and conducting malaria diagnoses at the community level | 39 |
| MVA | People's KAP towards Mass vaccine administration/R21 | 39 |
| MDA | People's KAP about MDA | 34 |
| Diagnosis malaria | Malaria diagnosis through the use of Rapid Diagnostic Tests (RDTs). | 33 |
| Pharmacy | Local drug shop where most of the people go to buy medicine without doctor’s prescription | 33 |
| Symptoms of malaria | Community knowledge regarding the signs and symptoms of malaria | 32 |
| Economic condition | People's financial capacity to afford treatment costs and other related expenditures | 31 |
| Causes of malaria | What are the causes of malaria. | 29 |
| Cost of treatment | What is the cost of treatment, and how do individuals manage these expenses | 29 |
| Fear of side effects (vaccine) | People's fears and concerns regarding potential adverse reactions following vaccination | 24 |
| Livelihood | People's livelihoods, including their occupations, sources of income, and methods of earning money to afford medical treatment | 22 |
| Transportation | The methods transport and routes individuals use to reach healthcare facilities | 21 |
| Culture | Ethnic cultures, including their beliefs, practices, and rituals | 20 |
| Health facilities | An assessment of treatment accessibility and the healthcare facilities or providers where people seek medical care. | 20 |
| Quality of treatment | People's satisfaction on treatment and how good and effective the care is and which treatment is better | 19 |
| Education | Literacy rates and the community’s interest in education | 19 |
| Health awareness | The level of health education and the extent of individuals' interest in utilizing modern treatment methods | 19 |
| Malaria | People's existing knowledge about malaria | 18 |
| Fear of malaria | People's current concerns and levels of fear regarding malaria | 17 |
| Period of malaria | The periods during people are most commonly affected by malaria | 17 |
| Fear of law | Concerns and fears regarding government policies and regulations implemented during COVID-19 vaccination campaigns. | 14 |
| Hill medicines | Traditional treatment using herbal Medicines made from plants and natural resources found in hilly areas. | 14 |
| Diarrhea | The most prevalent diseases experienced by the population, which occasionally lead to outbreaks | 13 |
| Rumors | Various false and misleading information about the COVID-19 vaccine was disseminated during the pandemic | 13 |
| Fever | One of the common diseases | 10 |
| Period of illness | A type of fever that occurs seasonally. | 10 |
| Causes of diseases | Which factors causes of common disease | 9 |
| Hospital | Upazila Health Complexes and other government hospitals provide free medical services to the community | 8 |
| Household head | Who can take decision including where to go for treatment and responsible for managing family | 8 |
| Fear of blood test | Fear or anxiety related to blood sample collection for testing | 8 |
| Religious leader | Responsibility and possesses the authority to influence others. | 8 |
| Community clinic | The nearest healthcare facility and small local health centers that provide free primary healthcare services in villages | 7 |
| Mosquitos | People's knowledge about mosquito which mosquito contains malaria parasite | 6 |
| Virus fever | A type of fever that occurs seasonally. | 6 |
| Use of Technology | What types of technology are currently being used by people—such as television, radio, and mobile phones—for entertainment and accessing news | 6 |
| Fear of injection | Being scared or anxious about getting a shot or needle. | 6 |
| Severe malaria | What measures do community people or healthcare providers undertake to manage cases of severe malaria | 6 |
| EPI worker | Who serves as the vaccinator for the Expanded Program on Immunization within communities | 6 |
| Gastric | Stomach-related illnesses and other common diseases often arise due to unhealthy or improper dietary habits. | 5 |
| Traditional law | As a barrier to accessing treatment, and people have their own low | 5 |
| Antibiotics | People are frequently using antibiotics without a doctor's prescription | 5 |
| Vaccine hesitancy | People's reluctancy to receive the COVID-19 vaccine and many did not complete the full vaccination schedule | 5 |
| Youth | Youth is also considered an influential person to their community and they can share information among their villagers | 4 |
| Migration | Some people have migrated and settled in the Lama and Alikadam areas in search of better livelihood opportunities, safety, or improved living conditions. | 4 |
| First aid | The immediate steps or measures people take when they become ill. | 3 |
| Typhoid | Kind of fever and common disease | 3 |
| Dev Care Foundation | The level of knowledge within the community regarding Dev Care Foundation, a local NGO | 3 |
| Politics | Local armed groups that claim to advocate for freedom but engage in extortion and oppress ordinary citizens. | 3 |
| Settlers | Impacts of settlement in the Hill Tracts. | 2 |
| Coughs | One of the common diseases | 1 |
| Religion | Religious diversity among Tripura community | 1 |
| Teacher | Teachers are also considered influential figures within communities and play an important role in sharing information. | 1 |
